# A Novel Look at Mechanisms and Applications of Xanthohumol (XN) in Dermatology and Cosmetology

**DOI:** 10.3390/ijms252211938

**Published:** 2024-11-06

**Authors:** Anna Kołodziejczak, Marta Dziedzic, Barbara Algiert-Zielińska, Paulina Mucha, Helena Rotsztejn

**Affiliations:** 1 Chair of Cosmetology, Department of Cosmetology and Aesthetic Dermatology, Faculty of Pharmacy, Medical University of Lódź, Muszyńskiego 1 Street, 91-151 Łódź, Poland; anna.kolodziejczak@umed.lodz.pl (A.K.); barbara.algiert@umed.lodz.pl (B.A.-Z.); helena.rotsztejn@umed.lodz.pl (H.R.); 2 Chair of Cosmetology, Department of Cosmetic Raw Material Chemistry, Faculty of Pharmacy, Medical University of Lódź, Muszyńskiego 1 Street, 91-151 Łódź, Poland

**Keywords:** xanthohumo, antioxidant, chalcone, tyrosinase inhibitor, acne, skin hyperpigmentation, hop extract, aging, dermatoses, skin ageing

## Abstract

Xanthohumol (XN), representing the group of chalcones, is a hydroxyl and superoxide free radical scavenger. It also has antimicrobial properties, showing antibacterial activity against Staphylococcus aureus, Staphylococcus pyogenes, Staphylococcus epidermidis and Propionibacterium acnes. XN exerts an inhibitory effect on tyrosinase (it hinders the oxidation of l-tyrosine and l-DOPA). However, it also affects the transport of pigment (through a reduction in the number and length of dendrites) and its degradation (through damage to melanosomes). Additionally, it has been shown to inhibit the different activation pathways of the premeditated response in macrophages and reduce the secretion of pro-inflammatory cytokines TNF-α, IL-6 and IL-1β. Xanthohumol also improves skin elasticity by reducing the activity of elastase and MMP 1, 2 and 9, and it increases the expression of type I, III and V collagen, as well as elastin and fibrillins in skin fibroblasts. It acts against the main factors contributing to the pathogenesis of acne by inhibiting pro-inflammatory mediators (e.g., COX-2, PGE2, IL-1β and TNF-α). Moreover, it shows antibacterial activity against *P. acnes* and *S. aureus*, as well as seboregulatory and antioxidant properties. It has also been recognized that XN intake could affect diabetic wound healing. XN shows antitumoral activity, e.g., in the case of skin melanoma, which is associated with the antioxidant, pro-apoptotic, anti-angiogenic and immunostimulating effects of this compound.

## 1. Introduction

**Scheme 1:**
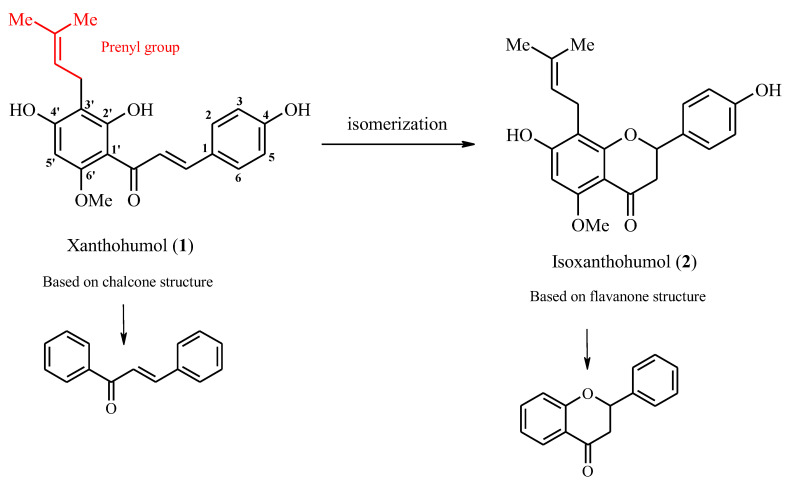
Isomerization of xanthohumol (**1**) to isoxanthohumol (**2**).

Representing the group of chalcones (intermediates in the biosynthesis of flavonoids), Xanthohumol (XN) (2′,4,4′-Trihydroxy-6′-methoxy-3′-(3-methylbut-2-en-1-yl)chalcone) (1) (Scheme 1) is a yellow, odorless powder, insoluble in water. However, it is well soluble in alcohols (e.g., methanol, ethanol) and alcohol–water mixtures, carboxylic acid esters (e.g., ethyl acetate), ethers (e.g., diethylether), ketones (e.g., acetone) or alkyl halides (e.g., chloroform). Under standard conditions, xanthohumol remains chemically stable.

This compound is derived from hop cones and is the predominant component of hops, making up 0.1–1% of their dry weight and accounting for 80–90% of the total content of prenylated hop flavonoids. In addition to chalcones containing prenyl groups (marked red in Scheme 1), this group also includes the corresponding flavanones [1,2]. The hydroxyl group in the C-2′ position of xanthohumol, which is based on the chalcone structure, may participate in ring cyclization reactions. Therefore, it is easily isomerized to a flavanone structure of isoxanthohumol (2) (Scheme 1).

So far, chemical methods have most often been used for the preparation of xanthohumol, such as synthesis using 2,4,6-trihydroxyacetophenone as a precursor, or the isolation, by multistage chromatography, of extracts from natural raw materials on silica gels with appropriate solvents. However, these processes are complicated and time-consuming, and the total amount of the compound obtained in this way is relatively small. A newer method of xanthohumol extraction and purification is based on submitting extracts from female hops to high-speed countercurrent chromatography (HSCCC), in which the absence of a solid adsorbent prevents the irreversible binding of a large part of the purified product to the substrate [3,4]. Xanthohumol, due to its wide spectrum of biological activity, arouses interest in many industrial fields. It shows, among others, strong antioxidant, anti-inflammatory, antifungal and antibacterial properties [4,5,6]. A limitation in the applicability of xanthohumol in certain areas may result from its low solubility in water. Polarity can be increased by complexation using cyclodextrins or glycosidic biotransformations [6]. Its isomer, isoxanthohumol, is more soluble, but much less biologically active (Scheme 1) [3].

## 2. Effect on the Skin 

### 2.1. Antioxidant Properties

Reactive oxygen species (ROS) exacerbate numerous inflammatory conditions throughout the body. Skin inflammation resulting from oxidative tissue damage (excess ROS) is associated not only with acne, but also with skin aging (photoaging and inflammation), as well as cardiovascular diseases, autoimmune disorders and cancer. Therefore, products with antioxidant potential have found widespread use in medicine, diet therapy and the cosmetics industry. The antioxidant activity of hop extracts is commonly assessed using well-established methods, such as DPPH (2.2-diphenyl-1-picrylhydrazyl radical scavenging assay), ORAC (oxygen radical absorbance capacity), TEAC (Trolox equivalent absorbance capacity; 6-hydroxy-2,5,7,8-tetramethylchromane-2-carboxylic acid, a hydrophilic derivative of tocopherol), ABTS (2.20-azino-bis-(3-ethylbenzothiazoline-6-sulphonic acid) radical scavenging assay and FRAP (ferric reducing antioxidant power). In a study by Inui et al., xanthohumol was found to exhibit the highest activity in anti-NO production assays [7].

The antioxidant capacity of flavonoids is strongly dependent on their chemical structure and the substrate/environment in a given product. Wu et al. demonstrated that ethanolic extracts showed better antioxidant effects than hot water extracts in TEAC, DPPH and reducing power assays, which were attributed to higher contents of total flavonoid and/or phenolic compounds [8,9]. Żołnierczyk et al. examined the antioxidant properties of xanthohumol and its derivatives, aiming to determine whether the acylation of one or two of the three hydroxyl groups in XN (1) would not reduce the antioxidant activity compared to the starting substrate. Compared to XN (1), 4-*O*-acetyl xanthohumol (2) showed twice the antioxidant activity in the DPPH assay, with xanthohumol exhibiting greater potency (stronger antioxidant) than the standard antioxidant, Trolox [3]. The study also showed that xanthohumol is a stronger antioxidant than the standard Trolox [3].

Gerhauser et al. proved that XN is a nine times stronger scavenger of hydroxyl and a three times stronger scavenger of peroxide free radicals than Trolox at a concentration of 1 μm on the ORAC test. Xanthohumol also has superoxide anion scavenging properties [10].

Wang’s research demonstrated that hop cone extracts rich in polyphenols, such as xanthohumol, provided both in vivo and in vitro protection against mutagenesis and oxidative damage, with similar effects to those of green tea polyphenols, at the same concentration [11].

The antioxidant activity of the hop extract was also tested in vivo by Yamaguschi et al. using the ORAC method. The result obtained for xanthohumol was significantly higher than that for vitamins C and E, and comparable to polyphenone 60 (used as a control), which has the highest ORAC values among edible plants. The authors also observed significantly higher SOAC values, i.e., the ability to quench singlet oxygen, for xanthohumol, obtaining values that were 8–15 times higher for it compared to polyphenone 60 or vitamin E. Xanthohumol was determined to have the highest activity regarding total oxygen radical absorption and singlet oxygen absorption. Considering the role singlet oxygen plays in exacerbating skin dermatoses (e.g., acne, atopic dermatitis or skin aging), the obtained result is promising from the dermatological and cosmetological perspectives. Collectively, these data suggest that xanthohumol could serve as a well-balanced antioxidant, with activity in both fat- and water-soluble environments, similar to the combined effects of vitamins C and E [4].

### 2.2. Antimicrobial Properties

As a result of the wide biological properties of xanthohumol, studies on its antimicrobial properties have been conducted. It has been shown that XN inhibits the growth of bacteria, fungi and viruses. Several studies have reported the anti-biofilm and antibiotic properties of xanthohumol. Studies on the antibacterial activity of XN prove the inhibitory effect preventing the growth of the Gram-positive bacteria *g. Staphylococcus aureus*, *Staphylococcus pyogenes* and *Staphylococcus epidermidis*, the skin pathogens *Propioni bacterium acnes* and the *Streptococcus mutant* which causes caries [4,12,13]. Concurrently, an in vivo study on rats showed that, after the administration of xanthohumol, no changes in the composition of the intestinal bacterial flora were observed. Pure xanthohumol was slightly more active against *S. aureus* strains (MIC range 15.6–62.5 μg/mL) than an extract of hop cones containing 51% xanthohumol w (MIC range 31.2–125.0 μg/mL) [13]. The antifungal properties of XN include the inhibition of two *Trichophyton* spp. fungi [6].

### 2.3. Effect on Skin Pigmentation

Kim et al. [14] investigated the tyrosinase-inhibiting properties of mushrooms, a methanol extract of Humulus lupulus L., and flavonoids isolated from it, including xanthohumol. They confirmed the strong tyrosinase-inhibiting activity of both the extract and individual flavonoids, suggesting their potential use in lightening hyperpigmented skin patches. Among all the tested compounds, xanthohumol exhibited the most potent effect, effectively inhibiting the oxidation of l-tyrosine and l-DOPA. Its potency proved to be comparable to that of kojic acid, which was the control sample. These studies were conducted on mushroom tyrosinase, so the expected effect in cosmetic formulas may differ from that obtained in the above study. 

Goenka et al. assessed the effect of xanthohumol (>98% purity) on melanogenesis in both melanoma cells and healthy skin cells in vitro. Xanthohumol was found to inhibit melanin transport by reducing the number and length of dendrites and by inducing melanosome degradation in HaCaT cells. The authors concluded that xanthohumol is a promising compound as an inhibitor of skin pigmentation focused on the transport of the pigment, but also its degradation, without affecting the synthesis of melanin (mechanism of reduced dendricity—a new mechanism of action in melanocytes for XN). XN may induce the degradation of melanosomes in keratinocytes, which in turn may contribute to the reduction in the melanin content in keratinocytes. The researchers additionally showed that xanthohumol inhibited melanogenesis in human MNT-1 melanoma cells, but also, unexpectedly, stimulated cellular tyrosinase activity. The authors highlighted that xanthohumol likely inhibits pigmentation through alternative distal pathways beyond tyrosinase inhibition [15]. Given its antioxidant properties, xanthohumol may also be beneficial in cosmetology as a supportive treatment for skin hyperpigmentation. 

### 2.4. Anti-Inflammatory Effect

Studies on the anti-inflammatory activity of xanthohumol have shown that it inhibits various paths of activation of the inflammatory response in macrophages, i.e., in LPS-activated macrophages, it inhibits NK-kB transactivation by reducing the expression of TLR4 and MD2, and in activated IFN-γ, it inhibits STAT-1α and IRF binding activity-1 [16]. Cho et al. conducted a survey on the effect of XN on the production of IL-12 (a cytokine affecting the differentiation of Th1 in the immune response), which is important in inflammatory skin diseases with dominance of Th1. The authors of the study used a model of chronic dermatitis induced by oxazolone in the mouse ear as an experimental model of psoriasis. They demonstrated the anti-inflammatory effect of XN in olive oil with acetone against chronic allergic dermatitis (CAD). The researchers then prepared an ointment using sorbitan sesquioleate, Tween 80, paraffin liquid and Vaseline, which also inhibited the growth of ear thickness after oxazolone induction. They emphasized the need to improve the formula to increase the penetration of XN and, thus, to improve the anti-inflammatory properties. These results confirm the validity of the topical use of preparations with xanthohumol in inflammatory skin diseases [17].

During the inflammatory response, pro-inflammatory cytokines, such as TNF-α, IL-1β and IL-6, are released and are involved in the development of various inflammatory skin diseases. Therefore, inhibition of these cytokines is crucial for suppressing inflammation. Hop extracts have been evaluated for their anti-inflammatory activity and have demonstrated the ability to reduce nitric oxide (NO) production and the secretion of pro-inflammatory cytokines TNF-α, IL-6 and IL-1β in vitro [8].

The impact of xanthohumol on the modulation of interleukin (IL)-12 production has also been studied, confirming its significant and extensive anti-inflammatory effects. Xanthohumol exhibited the strongest inhibition of IL-12 production compared to other related compounds. An in vivo study also demonstrated the clinical anti-inflammatory effects of XN, i.e., a reduction in chronic allergic contact dermatitis. It was concluded that the compound can be effectively used as an anti-inflammatory agent to reduce skin inflammation, and it works by inhibiting the production of IL-12, the most important factor driving T helper 1 immune responses [17].

### 2.5. Anti-Aging Effect on the Extracellular Matrix

Xanthohumol also improves skin elasticity by inhibiting the activity of elastase and MMP 1, 2 and 9. Additionally, it increases the expression of type I, III and V collagen, as well as elastin and fibrillins in skin fibroblasts [18]. Yamaguchii et al. showed that xanthohumol showed the highest activity against interstitial collagenase (MMP-1) and nutrophil collagenase (MMP-8) among seven naturally derived components from hop plant (*Humulus lupulus* L.) extracts. MMPs’ degradative aging processes, e.g., their increased expression of collagenases MMP-1 and -8, play a major role in the breakdown of type 1 collagen in the skin. The activity of MMPs, which degrade the extracellular matrix, is also important in the pathogenesis of acne. Therefore, in the treatment of acne, it is recommended to use compounds that are also aimed at the reconstruction of the extracellular matrix in the skin [4]. Philips et al. have shown that XN can inhibit the activities of MMPs (MMP-1, 2 and 9) and elastase. They also assessed significant effects on the increase in protein and/or transcription levels of collagen types I, III and V, as well as elastin and fibrillin 1 and 2 in skin fibroblasts. The effects were similar to those of ascorbic acid. These actions indicated a role in skin aging reversal [18]. 

### 2.6. Anti-Acne Effect

Both acne and skin aging are influenced by mechanisms involving singlet oxygen. For example, the peroxidation of squalene during sun exposure is mainly caused by singlet oxygen and free radical attack. Moreover, in people with severe forms of acne, increased concentrations of superoxide anions and the dysfunction of intracellular antioxidant mechanisms have been observed. Furthermore, *P. acnes* promotes inflammation by inducing the production of IL-6 and oxidative stress [19]. The antioxidant activity of xanthohumol can be widely used in the treatment of inflammatory skin diseases. It is believed that the most effective acne therapy is achieved through a multidirectional approach, including antioxidant, anti-inflammatory, antibacterial, keratolytic and seboregulating actions. The results presented in the study by Yamaguchi et al. confirm that xanthohumol acts against the main biological factors responsible for the pathogenesis of acne, i.e., it inhibits pro-inflammatory mediators (such as COX-2, PGE2, IL-1β and TNF-α) triggering skin inflammation [4]. *P. acnes* releases lipases, proteases and hydrolases into the sebum, which promotes inflammation and tissue destruction, also by the oxidative mechanism. Degraded hyaluronic acid activates TLR-2 in keratinocytes of the hair follicles, leading to the production of pro-inflammatory cytokines (e.g., IL-6, IL-1β, TNF-α or IL-8). Weber et al. conducted a study on 0.3% hop extract used in a botanical gel, together with other botanical actives, such as salicylic acid, Salix daphnoides bark extract, Gentiana lutea root extract, Leptospermum scoparium branch/leaf oil (Manuka oil) and Mentha arvensis herb oil, including preservatives such as sodium levulinate and sodium anisate. They showed that the gel formulation with the 0.3% hop extract had an antibacterial effect against *P. acnes* (5.5 mm zone of inhibition) and *S. aureus* (3 mm zone of inhibition), which was significantly better than the placebo gel. The positive control, clindamycin antibiotic gel, exhibited a zone of inhibition measuring 9 mm. Based on the results, it was concluded that due to its antioxidant, anti-inflammatory and antibacterial properties, a hop extract (e.g., xanthohumol) may be used as a supplementary treatment for acne. It was also shown that the tested hop extract, following solar irradiation of human primary keratinocytes, showed a decrease in IL-6 expression [19]. Yamaguchi et al. have also shown that xanthohumol has antibacterial activity against *P. acnes* [4]. Weber et al. conducted a study to evaluate the degreasing effect and skin tolerance of a facial cleanser, which contained, among others, a hop extract, and a control preparation of plain SLS. The sebum analysis showed that the product with xanthohumol decreased the level of sebum in a statistically significant way (*p* < 0.01). None of the preparations caused skin redness (which was measured with the Mexameter) [20]. Therefore, based on the antioxidant, antimicrobial, anti-inflammatory and depigmenting properties demonstrated in these studies, xanthohumol may be considered a promising ingredient with great potential for use in cosmetology. Adamiak concluded, from Yamaguchi’s research [4], that XN can be used for the reduction of aging symptoms, photoprotection and in dermatology in the treatment of acne, atopic dermatitis, cancer and skin pigmentation disorders [21].

### 2.7. Protective and Accelerating Wound Healing Properties

In 2022, Adamiak et al. conducted a study evaluating the influence of xanthohumol on the physicochemical properties of fish skin collagen films before and after UV irradiation. The UV sterilization of materials can lead to collagen film degradation. Collagen irradiated by UV light exhibits a loss of the triple helical structure. Infrared spectroscopy, atomic force microscopy (AFM) and contact angle and mechanical measurements were used for observation and evaluation. Previous studies have shown that collagen peptides, when used as biomaterials, protect against photodamage to human skin fibroblasts and inhibit MMPS (MMP-1 and MMP-9). Adamiak et al. showed that collagen films were more hydrophobic in the presence of xanthohumol, which was manifested by lower water loss after UV irradiation without XN. This led to an improvement in both the surface and the mechanical properties of the collagen biomaterial, specifically enhancing skin adhesion [21]. The study also evaluated whether XN intake could enhance diabetic wound healing due to its antioxidant and anti-inflammatory potential, along with improved neovascularization. A study was conducted on healing the skin of rats with type 1 diabetes (Table 1). XN was shown to reduce the recruitment and activation of inflammatory cells, which contributed to the resolution of the inflammatory phase and facilitated progression to the next stages of healing. Consuming beer enriched with XN increased systemic levels of reduced glutathione (GSH) [22].

### 2.8. Antitumoral Activity

Fonseca et al. developed a PLGA (poly-lactic-co-glycolic acid) nanoparticle containing 90% xanthohumol and tested it against malignant skin melanoma in mice. XN exhibits poor bioavailability, low water solubility and stability, high photosensitivity, and a short half-life. Polymeric nanoparticles are good carriers to load xanthohumol as they are FDA-approved and are excellent transporters for drug encapsulation. The final anti-melanoma effect results from xanthohumol cytotoxicity and inhibition of tumor cell proliferation and migration. This study indicates progress toward a nanoformulation for delivering xanthohumol to reduce side effects associated with chemotherapeutic drugs. The authors also emphasize that the preparation may produce a preventive effect against malignant skin melanoma [23].

Seitz et al. conducted an in vitro study on cell culture with human melanoma cells, showing that xanthohumol is an inhibitor of a series of pro-cancer reactions that lead to melanoma metastasis. They also conducted a study on a mouse model of melanoma metastasis and found that XN inhibited the growth of metastases in the liver. The study revealed significantly larger areas of central necrosis compared to control mice [24]. The anti-cancer effect is essentially related to the pro-apoptotic, anti-angiogenic and immunostimulating properties of the active substances. Modulation of autophagy, a nonspecific protein degradation pathway, significantly contributes to XN’s antitumor activity. Additionally, XN has the ability to inhibit the enzymatic pathways involved in the proliferation and migration of endothelial cells [25]. XN is an inhibitor of the initiation, promotion and progression of carcinogenesis phases. Thus, it has an exceptionally broad spectrum of inhibitory activity in carcinogenesis. The compound suppresses cytochrome P450 activity and demonstrates anti-inflammatory properties by inhibiting the activity of cyclooxygenase (COX)-1 and COX-2, as well as the production of nitric oxide. It prevents the proliferation of tumor cells by inhibiting DNA synthesis and blocks angiogenesis, which is essential for solid tumor growth and dissemination [16].

The anti-apoptotic effect cannot be omitted in the supplementary characterization of xanthohumol activity. The primary mechanism by which XN inhibits apoptosis is through modulation of the expression of caspase-3, -7, -8 and -9, which are pro-apoptotic factors [25].

## 3. Other Effects and Properties of Xanthohumol

Dietary hop extracts rich in XN exhibit antiviral and hepato-preventive properties, mainly by protecting the liver from oxidative damage. In vivo studies on Tupaiabelangeri (shrew) by Yang et al. show that XN reduces hepatitis, fibrosis and inflammation in HCV-infected animals, mainly by inhibiting oxidative reactions and regulating apoptosis. Nevertheless, the authors believe that the anti-HCV potential of XN requires further research [26]. 

Xanthohumol also exhibits properties that block the oxidation of LDL (inhibiting the Cu2+-mediated oxidation of LDL), as demonstrated in a study by Miranda et al. The authors achieved a reduction in lipid peroxidation of over 70% after five hours of incubation compared to the control sample, inhibition of the oxidation of tryptophan residues in LDL and the formation of TBARS [27]. Rodriguez et al. confirmed the antioxidant effects of prenylated chalcones, including xanthohumol, on microsomal lipid peroxidation in the rat liver. XN may help to prevent weight gain by inhibiting fat absorption and fatty acid metabolism. Numerous studies have shown that xanthohumol has an effect on metabolic syndrome and related disorders. It has been proven that a high-fat diet enriched with XN in rats regulates the hepatic metabolism of fatty acids and inhibits fat absorption in the intestine, which, among other effects, prevents weight gain. The tested compound inhibits glucose uptake in intestinal cells and decreases the activity of α-glucosidase. It is also an inhibitor of diacylglycerol acyltransferase, involved in the synthesis of triglycerides [28]. 

In studies on mice, Zamozow et al. suggest that xanthohumol may play a role in improving cognitive function in young animals. However, it seems to be ineffective in older individuals [29]. Rancan L et al. demonstrate that xanthohumol has a protective role in secondary brain disorders associated with aging, also understood as protection against brain degenerative diseases [30]. Studies have shown a dose-dependent effect of XN on the balance between pro- and anti-apoptotic factors in the brain by limiting the excessive production of pro-inflammatory cytokines (IL 1 and TNF alpha) [25]. 

Recent studies also prove the potential effects of xanthohumol, isoxanthohumol and 8-prenylnaringenin related to the treatment of cancer. XN has a wide range of anti-cancer properties, fulfilling not only antioxidant and anti-inflammatory, but also anti-angiogenic and chemopreventive functions. It has been shown to be active against cancerous diseases such as skin melanoma, prostate cancer, hepatocellular carcinoma and breast cancer [24,31,32,33,34]. 

Research results indicate that XN inhibits osteoclastogenesis and may be useful in preventing bone diseases. Additionally, XN may be a natural compound hindering hyaluronan overproduction and subsequent osteoarthritis [35]. 

Results have shown that XN stimulates the uptake of iodide in rat thyrocyte cells. Additionally, it affects the metabolism and distribution of hormones [35]. It is also indicated that Humulus lupulus L. extract containing prenylated chalcone xanthohumol (XN) may be used in women in and after menopause. A study by van Breemen et al. demonstrates the long half-lives of the estrogenic and proestrogenic prenylated phenols in hops, with no acute toxicity [36]. An escalating-dose study was carried out in menopausal women to evaluate the safety of XN. The results show that this extract does not affect sex hormones or blood clotting, and reveals no acute toxicity [35].

A study was conducted in which glomerular endothelial and tubular epithelial cells were grown under the influence of high glucose stimulation. An in vitro model of diabetic nephropathy was developed. This was followed by using approximately 150 natural small-molecule drugs. Xanthohumol showed the best protective effect on the renal cells. Subsequently, the compound was used to treat kidneys in mice, and it was shown to improve kidney damage by alleviating oxidative stress through the Nrf2 signaling pathway. Xanthohumol also reduces the oxidative stress associated with type 2 diabetes. Additionally, other studies have shown that it improves the skin wound healing through its anti-free-radical, anti-inflammatory and angiogenesis-regulating effects in rats with type 1 diabetes [37]. 

XN’s antiviral activity was assessed against IV-1, BVDV, herpes (HSV-1, HSV-2, CMV), HCV and coronaviruses. XN was shown to inhibit cytopathic effects, viral p24 antigen production and reverse transcriptase activity in HIV-1-infected C8166 lymphocytes. Additionally, xanthohumol moderately inhibits HIV-1 replication in peripheral blood mononuclear cells. However, it has not been reported to decrease recombinant HIV-1 reverse transcriptase activity or HIV-1 entry [38]. Apart from HIV, XN also inhibits bovine diarrhea virus (BVDV), hepatitis C virus (HCV) replication in a cell culture system, comparable to that of IFN-α, and low-to-moderate herpes virus (HSV-1, HSV- 2, CMV) [39,40]. Studies on the antiviral activity of xanthohumol against coronaviruses have shown that it is a strong pan-inhibitor of various coronaviruses, e.g., the SARS-CoV-2 betacoronavirus (IC50 value 1.53 μM) and PEDV alphacoronavirus (IC50 value 7.51 μM), targeting the major protease (Mpro), which plays a key role in viral replication and transcription. Xanthohumol also inhibited the activity of Mpro in enzyme assays, while, when applied as pretreatment, it reduced SARS-CoV-2 and PEDV replication in Vero-E6 cells [41]. XN decreases HIV-1-induced cytopathic effects. It also inhibits BVDV, HSV-1, HSV-2 and CMV to a low-to-moderate extent, as well as HCV replication in cell culture systems, comparable to IFN-α [35]. 

## 4. Future Perspectives

Since xanthohumol is associated with its ability to reduce the expression of proinflammatory genes, both pro-inflammatory enzymes (e.g., COX) and cytokines, it may in the future be used interchangeably with steroid therapy or as an adjunct to the anti-inflammatory action of glucocorticoids. Inflammatory dermatosis is usually treated with glucocorticoids, but due to some side effects, in some cases not possible to use, e.g., in the case of chronic inflammatory skin diseases. It is in this area that the local application of XN shows promising potential. In the future, it will be possible to test and determine the appropriate concentration of the xanthohumol complex in the final specific formulation, allowing for therapeutic effects in the case of skin dermatoses associated with inflammation. Its application in cosmetology is extensive, including strengthening skin regeneration following barrier-disrupting treatments, such as needle mesotherapy, skin micro-needling, fractional laser therapy and chemical peelings. In these cases, the use of a xanthohumol complex may be important for rebuilding the antioxidant barrier, because the local demand for antioxidants increases as a result of inflammation. The use of XN may prevent local lipid peroxidation, which in turn leads to dysfunction of the entire lipid barrier. Strengthening the skin’s antioxidant system is of great importance in the case of UV-damaged, photoaging, sensitive and over-reactive skin exposed to stress. Xanthohumol complexes can be used in the treatment of rosacea and skin discoloration. An alternative application is the prevention of bacterial superinfections after ablative laser therapy. Xanthohumol can be used to increase the potential of other active ingredients in skin care products [5].

## 5. Conclusions

Xanthohumol exhibits potent antioxidant, anti-inflammatory and antimicrobial activities, making it a promising compound for use in anti-aging skincare and dermatological treatments. Therefore, the compound can be used as an active ingredient in anti-aging preparations and in dermatology as an auxiliary therapy in the anti-inflammatory treatment of dermatoses including, various forms of acne. Given these properties, xanthohumol offers substantial therapeutic value as an adjunct or alternative to conventional treatments, with potential applications extending into cosmetology and dermatology.

## Figures and Tables

**Table 1:** Summary of the main properties of xanthohumol in terms of the effect on the skin.

The Most Important Properties of Xanthohumol	Brief Characteristics of Action	References
antioxidant	scavenger of free hydroxyl radicals, superoxide radicals, superoxide anion radicals, nitric oxide	[3,4,7,8,9,10,11]
antimicrobial properties (anti-acne effect)	inhibition of the growth of *Staphylococcus aureus*, *Staphylococcus pyogenes*, *Staphylococcus epidermidis*, *Propionibacterium acnes*	[4,6,12,13,19]
antihyperpigmentation effect	tyrosinase-inhibiting properties, skin pigmentation inhibitor targeting pigment transport, induction of melanosome degradation in keratinocytes	[15]
anti-inflammatory effect	inhibition of inflammatory response activation pathways in macrophages, inhibition of IL-12 production in vitro ability to reduce NO production and secretion of proinflammatory cytokines TNF-alfa, IL-6 and IL-1	[8,16,17,22]
anti-aging effect on the extracellular matrix	inhibition of elastase activity and MMP 1, 2, 8 and 9 increase in expression of collagen types I, III and V and elastin and fibrillins in skin fibroblasts	[4,18,21]
